# Quality assessment of a clinical next-generation sequencing melanoma panel within the Italian Melanoma Intergroup (IMI)

**DOI:** 10.1186/s13000-020-01052-5

**Published:** 2020-12-14

**Authors:** Irene Vanni, Milena Casula, Lorenza Pastorino, Antonella Manca, Bruna Dalmasso, Virginia Andreotti, Marina Pisano, Maria Colombino, Italian Association for Cancer Research (AIRC) Study Group, Alessia Covre, Anna Maria Di Giacomo, Michele Maio, Francesco De Logu, Daniela Massi, Francesca Portelli, Andrea Anichini, Roberta Mortarini, William Bruno, Francesco Cabiddu, Francesco Spagnolo, Grazia Palomba, Maria Cristina Sini, Maria Antonietta Fedeli, Amelia Lissia, Ulrich Pfeffer, Enrica Teresa Tanda, Carla Rozzo, Panagiotis Paliogiannis, Antonio Cossu, Paola Ghiorzo, Giuseppe Palmieri, for the Italian Melanoma Intergroup (IMI), Corrado Caracò, Antonio Maria Grimaldi, Virginia Ferraresi, Mario Mandalà, Roberto Patuzzo, Pietro Quaglino, Paola Queirolo, Ignazio Stanganelli

**Affiliations:** 1 Genetics of Rare Cancers, IRCCS Ospedale Policlinico San Martino, L.go R Benzi, 10, 16132 Genoa, Italy; 2 Genetics of Rare Cancers, Department of Internal Medicine and Medical Specialties, University of Genoa, Genoa, Italy; 3 Unit of Cancer Genetics, National Research Council (CNR), Sassari, Italy; 4 Tumor Epigenetics, IRCCS Ospedale Policlinico San Martino, Genoa, Italy; 5 Medical Oncology, IRCCS Ospedale Policlinico San Martino, Genoa, Italy; 6 Department of Medical, Surgical, and Experimental Sciences, University of Sassari, Sassari, Italy

**Keywords:** Melanoma, Gene panel testing, Next generation sequencing (NGS), Somatic mutations, Quality controls, *BRAF*, Target therapy

## Abstract

**Background:**

Identification of somatic mutations in key oncogenes in melanoma is important to lead the effective and efficient use of personalized anticancer treatment. Conventional methods focus on few genes per run and, therefore, are unable to screen for multiple genes simultaneously. The use of Next-Generation Sequencing (NGS) technologies enables sequencing of multiple cancer-driving genes in a single assay, with reduced costs and DNA quantity needed and increased mutation detection sensitivity.

**Methods:**

We designed a customized IMI somatic gene panel for targeted sequencing of actionable melanoma mutations; this panel was tested on three different NGS platforms using 11 metastatic melanoma tissue samples in blinded manner between two EMQN quality certificated laboratory.

**Results:**

The detection limit of our assay was set-up to a Variant Allele Frequency (VAF) of 10% with a coverage of at least 200x. All somatic variants detected by all NGS platforms with a VAF ≥ 10%, were also validated by an independent method. The IMI panel achieved a very good concordance among the three NGS platforms.

**Conclusion:**

This study demonstrated that, using the main sequencing platforms currently available in the diagnostic setting, the IMI panel can be adopted among different centers providing comparable results.

**Supplementary Information:**

The online version contains supplementary material available at 10.1186/s13000-020-01052-5.

## Introduction

Malignant melanoma is one of the most aggressive, drug-resistant human cancers, and its incidence has risen persistently during the last few decades, particularly in the Caucasian population [1]. According to GLOBOCAN, more than 287,723 new cases of melanoma of the skin occurred worldwide in 2018 (1.6% of all cancers), with approximately 60,712 reported deaths (GLOBOCAN 2018) [2]. In 2020, it is estimated that around 377,000 new cancer cases will be diagnosed in Italy and, among them, 14,863 cases are expected to be melanomas (AIOM, AIRTUM, I numeri del cancro in Italia 2020, available at: https://www.fondazioneaiom.it/wp-content/uploads/2020/10/2020_Numeri_Cancro-pazienti-web.pdf). Several tumor suppressor genes and/or oncogenes have been reported to be involved in melanomagenesis [3–6]. Of great interest are the RAS-RAF-MEK-ERK, PI3K/PTEN and c-Kit pathways, since patients harboring activating mutations in *BRAF*, *NRAS* and *KIT* genes could benefit of target treatment options or tailored combinations of target- and immuno-therapies. The identification of variants predictive of response or resistance to systemic treatments is already recommended today for proper management of advanced melanoma and molecular testing is a priority in determining the course of therapy. Indeed, molecular testing for actionable mutations is mandatory in patients with advanced disease (unresectable stage III or stage IV, and highly recommended in high-risk resected disease stage IIc, stage IIIb–IIIc). In case of a BRAF-wild type tumor, NRAS and c-KIT (mucosal and acrolentigenous primaries) testing should be performed (Italian Association of Medical Oncology/AIOM Guidelines Melanoma - 2019, available at: https://www.aiom.it/linee-guida-aiom-melanoma-2019/; National Comprehensive Cancer Network/NCCN clinical practice guidelines in oncology: melanoma - 2019, available at: https://www.nccn.org/professionals/physician_gls/pdf/cutaneous_melanoma.pdf) [7].

Recent evidence provided by the use of Whole Exome and Whole Genome Sequencing (WES and WGS) pointed out the involvement of other genes in melanoma pathogenesis, suggesting the importance of screening multiple genes at the same time to better classify the three main molecular melanoma subtypes (BRAF^mut^, RAS^mut^, and non-BRAF^mut^ /non-RAS^mut^) [3–6, 8–16].

To date, various molecular strategies are available for mutational analysis of the *BRAF* gene, such as Sanger Sequencing (SS), real-time PCR, high-resolution melting analysis, Peptide Nucleic Acid (PNA)-mediated real-time PCR clamping, digital PCR, pyosequencing, and immunohistochemistry. Each technique is able to detect mutations on single genes per run with a specific sensitivity, specificity, and limit of detection [17–24]. At the beginning, Cobas 4800 BRAF V600 Mutation Test (Roche Molecular Systems) and THxID™-BRAF kit (BioMerieux, Inc.) were the only FDA-approved assays for BRAF V600E mutation and for BRAF V600E/V600K mutations in DNA samples extracted from Formalin-Fixed Paraffin-Embedded (FFPE) human melanoma tissue, respectively (http://www.fda.gov/companiondiagnostics) [25–27]. The advent of high throughput Next-Generation Sequencing (NGS) technology has revolutionized the understanding of cancer biology and improved personalized treatment strategies in a large variety of human cancers, including melanoma. Development and use of NGS targeted gene sequencing panels may represent an attractive method in hospitals and clinics, since they can simultaneously screen disease-related mutations in multiple several genes per run, thus reducing both reagents cost and DNA quantity necessary, with enough sensitivity and specificity to detect somatic variants with frequencies higher than 5%. In the clinical setting, the application of NGS targeted gene panels requires analytical validation to ensure the detection of somatic variants and high quality of sequencing results [28]. NGS methods for cancer -related genes testing have been rapidly adopted by clinical laboratories [29], but no consensus on the use of NGS tests and validation of a customize panel in clinical practice for melanoma are established in Italy, yet. A consensus was reported by the AIOM 2019 guidelines, but only for *BRAF* mutations (AIOM Guidelines for Melanoma - version 2019, available at: https://www.aiom.it/linee-guida-aiom-melanoma-2019/).

Here, we present the design and the mutational concordance between three different NGS platforms of a customized panel that analyzes target regions of 25 genes frequently mutated in melanoma, based on literature evidences [5]. By using three NGS platforms often available in the research and clinical centers, this multicenter study aims to develop quality controls to be adopted by IMI centers.

## Materials and methods

### Samples’ collection

We selected a total of 11 metastatic melanoma cancer cases, 5 treated at the IRCCS Ospedale Policlinico San Martino (Genoa, Italy) and 6 treated at the Unit of Cancer Genetics, National Research Council (CNR) (Sassari, Italy). Both centers have passed previous External Quality Assessment (EQA) tests conducted by both the Italian Association of Medical Oncology (AIOM) and The European Molecular Genetics Quality Network (EMQN). These procedures of quality assurance are actually widely recognized systems to assess the performance of a laboratory, allowing laboratories to demonstrate consensus with their peers and providing information on inter-method comparability.

All samples were FFPE tissues, except for two fresh frozen tumor samples. All tumor samples were evaluated by pathologists for the presence of adequate tumor cell content (≥70%). The clinical characteristics of the metastatic melanoma patients are reported in Table 1. All specimens had already been screened for the presence of *BRAF* codon 15 mutations by SS approach and Real Time PCR assay (PNAClamp™ BRAF Mutation Detection Kit; Panagene, Daejeon, Korea) or Therascreen™ BRAF Pyro assay (Qiagen, Valencia, CA) for molecular diagnostic purposes.

**Table 1:** Clinical characteristics of the metastatic melanoma patients

Sample ID	Sex	I	LN MTS cell tumor content (%)	Melanoma site	Primary tumor syze	Regional lymph node status	B	M	U	P	***BRAF*** Exon 15 mutation by SS	***BRAF*** Exon 15 mutation by additional method	***NRAS*** Exon 2–3 mutations by SS
**#1**	M	G	~ 70	left lower leg	pT3b	pN3	3.55	9	Y	n.a.	WT	PNAClamp™ BRAF Mutation Detection Kit: WT	NM_002524: c.182A > G p.Gln61Arg
**#2**	F	G	> 90	left upper leg	pT4b	pN3	5.53	11	Y	Y	NM_004333: c.1799 T > A p.Val600Glu	PNAClamp™ BRAF Mutation Detection Kit: Mutated	n.d.
**#3**	M	G	> 80	right lower leg	n.a	pN3	2.92	3	n.a	N	NM_004333: c.1799 T > A p.Val600Glu	PNAClamp™ BRAF Mutation Detection Kit: Mutated	n.d.
**#4**	F	G	80–85	right arm	pT4a	pN3	12.5	14	Y	Y	WT	PNAClamp™ BRAF Mutation Detection Kit: WT	NM_002524: c.181C > A p.Gln61Lys
**#5**	F	G	~ 80	left upper leg	pT3a	pN3	3.5	5–9	N	Y	NM_004333: c.1799_1800delTGinsAC p.Val600Asp	PNAClamp™ BRAF Mutation Detection Kit: Mutated	WT
**#6**	M	S	~ 80	upper back	pT3a	pN2b	2.75	4	N	N	NM_004333: c.1799 T > A p.Val600Glu	Therascreen™ BRAF Pyro Kit: Mutated	WT
**#7**	M	S	> 80	left upper leg	pT3b	pN3b	3.53	5	Y	Y	NM_004333: c.1798_1799delGTinsAA p.Val600Lys	Therascreen™ BRAF Pyro Kit: Mutated	WT
**#8**	F	S	~ 80	left forearm	pT3a	pN2b	2.26	3	N	N	NM_004333: c.1799 T > A p.Val600Glu	Therascreen™ BRAF Pyro Kit: Mutated	WT
**#9**	M	S	> 80	right lower leg	pT4b	pN3b	7.45	8	Y	N	WT	Therascreen™ BRAF Pyro Kit: WT	WT
**#10**	F	S	~ 90	left foot	pT3b	pN2b	2.15	2	Y	Y	NM_004333: c.1790 T > G p.Leu597Arg	Therascreen™ BRAF Pyro Kit: Not Detected	WT
**#11**	M	S	~ 80	upper back	pT3a	pN2b	2.84	2	N	N	NM_004333: c.1799 T > A p.Val600Glu	Therascreen™ BRAF Pyro Kit: Mutated	WT

*Abbreviations*: *M* Male; *F* female; *I* institute; G: IRCCS Ospedale Policlinico San Martino, Genoa; S: Unit of Cancer Genetics at the National Research Council/CNR, Sassari; *LN MTS* lymph node metastases; *B* Breslow; *M* mitosis/mm2; *U* ulceration; *P* pigmentation; *SS* sanger sequencing; *Y* yes; *N* no; *n.a.* not available, *n.d.* not done, *WT* wild type

All patients were informed about the use of their tumour tissues samples for mutation analyses, gave the permission to collect tissue specimens for such purposes and signed a written consent. The study was approved by local Ethics Committees of the institution involved in this study (National Research Council and Ospedale Policlinico San Martino). Medical records were used for collecting clinical and pathological data (clinical presentation, tumour size and characteristics; Table 1).

### DNA extraction and quality control

Five genomic DNA (gDNA) samples from IRCCS Ospedale Policlinico San Martino were extracted from the tumor sections using the Genomic DNA FFPE One-Step Kit for Diatech MagCore® HF16Plus extractor (RBC Bioscience, New Taipei City, Taiwan) according to the manufacturer’s instructions. Quantity and purity of the gDNA was examined by SPECTROstar Nano (BMG Labtech, Offenburg, Germany) to measure the whole absorption spectrum (220–750 nm) and calculating absorbance ratios at both 260/280 and 260/230. Six gDNAs from Institute of Biomolecular Chemistry (ICB), National Research Council (CNR) were extracted from FFPE tissue sections with QIAamp DNA Mini purification kit and QIAamp DNA FFPE Tissue kit (Qiagen, Valencia, CA). DNA purity and concentration were assessed with both Nanodrop 2000 spectrophotometer (Thermo Scientific, Wilmington, DE, USA) and Qubit® 2.0 Fluorometer (Invitrogen, Carlsbad, CA, USA). Moreover, all samples were quantified by Qubit® 2.0 Fluorometer (Invitrogen, Carlsbad, CA, USA) and Agilent 2200 TapeStation system using the Genomic DNA ScreenTape assay (Agilent Technologies, Santa Clara, CA, USA). gDNA fragmentation status was evaluated by the Agilent 2200 TapeStation system using the Genomic DNA ScreenTape assay (Agilent Technologies, Santa Clara, CA, USA) able to produce a DNA Integrity Number (DIN). gDNA quality showed a DIN ranging from 2.9 to 8.6.

All DNA samples belonging to each laboratory were distributed in a blind-coded manner to the other.

### Melanoma panel design

The “IMI Somatic Panel” - IAD79062 - was created to facilitate the identification of the genetic regions most significantly associated with melanoma using the Ion AmpliSeq™ Designer™ tool [at https://ampliseq.com/login/login.action]; the chosen targets of 35.13 kb were entered into the online tool and the resulting 343 amplicons (ranging from 125 to 175 bp) were divided by the online designer into three primer pools to maximize target specificity [30].

### Targeted next generation sequencing (NGS)

All gDNA samples were blindly analyzed by both laboratories (IRCCS Ospedale Policlinico San Martino and Unit of Cancer Genetics at the National Research Council/CNR), using three different NGS platforms. The IRCCS Ospedale Policlinico San Martino center performed NGS analysis with the MiSeq™ Illumina and PGM™ Ion Torrent platforms, whereas the CNR center used the Proton™ Ion Torrent platforms. The DNA was amplified using the designed “IMI Somatic Panel” (3 primers pool), which analyzes 343 amplicons in target regions of 25 genes: *ARID2* (all coding sequences), *BAP1* (all coding sequences), *BRAF* (exons 1 and 15), *CCND1* (all coding sequences), *CDK4* (exons 1, 3 and 4), *CDKN2A* (all coding sequences), *DDX3X* (exons 2–3, 6–7, 10–15 and 17), *ERBB4* (exons 2–3, 8–12, 14, 21, 23, and 27), *GNA11* (exon 5), *GNAQ* (exon 5), *HRAS* (all coding sequences), *KDR* (Q472H), *KIT* (exons 2, 9–11, 13–15, and 17–18), *KRAS* (all coding sequences), *MAP2K1* (all coding sequences), *MET* (exons 1, 10, 13, 15 and 18), *MITF* (E318K), *NF1* (exons 28–30, 33–34, 36–37, 39, 41–43, 45, 48–53, and 55–58), *NOTCH1* (exons 26–27, and 34), *NRAS* (all coding sequences), *PIK3CA* (exons 1, 4, 6–7, 9, 13, 18, and 20), *PPP6C* (exons 2 and 4–7), *PTEN* (exons 1, 3, 5, and 8), *RB1* (exons 4, 6, 10–11, 14, 17–18, and 20–22), and *TP53* (exons 1, 3–7, and 9).

#### Illumina

Overall, 30 ng of gDNA for each sample was used for library construction using IMI Somatic Panel (3 primers pool) and Ampliseq Library PLUS for Illumina (Illumina Inc., San Diego, CA, USA) following the manufacturer’s instructions. Cycling conditions were performed according to the DNA type and primer pairs per pool: 23 cycles with an extension time of 4 min in the first multiplex PCR, whereas in the second, optional PCR, the gDNA were subjected to seven cycles. Sample libraries was combined and diluted to 2 nM, denatured with 0.2 N fresh NaOH, diluted to 8.4 pM by addition of Illumina HT1 buffer. Then, the libraries, spiked with 1% PhiX (8.4 pM), were sequenced on an Illumina MiSeq™ instrument by using the 300-cycle (2 × 150 paired ends) MiSeq v2 Reagent Kit v2 (Illumina).

#### PGM™ ion torrent

gDNA from the 11 tumor samples were amplified using the Ion AmpliSeq™ Library Kit 2.0 (ThermoFisher Scientific) starting from 30 ng of gDNA, barcoding each sample following the manufacturer’s instructions. Cycling conditions were performed according to the DNA type and primer pairs per pool: 23 cycles with an extension time of 4 min in the first multiplex PCR, whereas in the second, optional PCR, the gDNA were subjected to five cycles. The library size was checked using the Agilent High Sensitivity DNA Kit by the Bioanalyzer 2100 instrument (Agilent Technologies), and library concentration was evaluated with a Qubit® 2.0 Fluorometer using the Agilent High Sensitivity DNA Kit (Life Technologies). Each diluted library (100 pM) was amplified through emulsion PCR using the OneTouch™ Instrument (ThermoFisher Scientific) and enriched by the OneTouch™ ES Instrument (ThermoFisher Scientific) using the Ion PGM™ Hi-Q™ View OT2 Kit, following the manufacturer’s instructions. Finally, sequencing was performed on the Ion PGM™ (ThermoFisher Scientific) with the Ion PGM™ Hi-Q™ View Sequencing Kit (ThermoFisher Scientific), loading barcoded samples into a 316v.2 chip.

#### Proton™ ion torrent

The eleven libraries were generated starting from 30 ng of input DNA with the Ion AmpliSeq Library Kit 2.0, according with the manufacturer instructions, barcoded with Ion Xpress Barcode Adapters, diluted at a final concentration of 50 pM, and pooled together. Template preparation and chip loading were performed on the Ion Chef; PI™ v2 BC chips were subsequently sequenced on the Ion Proton™ instrument using the Ion PI™ IC 200 Kit.

### Bioinformatics analysis

The Variant Caller (VC) analysis for each samples was carried out using the Ion and Illumina informatics solution integrated by each specific NGS platform.

For Ion Torrent platforms, initial variant calling from the Ion AmpliSeq™ sequencing data was generated using Torrent Suite v.5.10.1 (ThermoFisher Scientific) with a plug-in VC program (VC v.5.10.1.20) with Generic - PGM (3xx) - Somatic - Low Stringency parameters. Moreover, Ion Reporter™ Software were used for variant annotation.

Illumina data was analyzed using BaseSpace (Illumina) to convert *.bcl files into FASTQ files, which contain base call and quality information for all reads passing filtering. DNA Amplicon App v.2.1.0 was used for alignment in the targeted regions (specified in a manifest file), or the Burrows Wheeler Aligner across the entire genome. We selected the option “Somatic Variant Caller” with a Variant Allele Frequency (VAF) threshold of 0.01 (Percentage) and a depth threshold of 10. The tertiary analysis was carried out using BaseSpace Variant Interpreter.

All identified variants were confirmed by the Integrative Genomics viewer (IGV) by visually examining mutations using Integrative Genomics Viewer software (http://www.broadinstitute.org/igv) [31].

### Sanger Sequencing (SS) validation

All NGS variants with frequency higher than 10% were validated by SS using primer sets, designed by Primer3Plus tool (http://www.bioinformatics.nl/cgi-bin/primer3plus/primer3plus.cgi). All primer sequences are reported in Table 2. The PCR reactions were performed by amplifying 40 ng of gDNA in a final volume of 15.5 μL containing 200 mol/L dNTPs, 10× Taq buffer, 0.322 μM of each PCR primer, 1.5 U of Taq Hot Start (Qiagen). The PCR program consists of 10 min at 95 °C and 35 cycles with 30 s at 95 °C, 30 s at specific annealing temperature of primer, and 30 s at 72 °C, followed by 5 min at 72 °C. Purified products were sequenced, using the same primers of the PCR amplification, with the BigDye Terminator v1.1 cycle sequencing kit (Applied Biosystems) under the following conditions: 1 μl BigDye Terminator v1.1, 2 μl sequencing buffer 5X, 3.2 pmol forward or reverse primer, 1.5 μl PCR purified product and 4 μl sterile water to a final reaction volume of 10.5 μl. Cycle sequencing was performed using initial denaturation step at 96 °C for 10 s followed by 25 cycles at 96 °C for 10 s, 60 °C for 3 min on GeneAmp® PCR System 9700 (Applied Biosystems). The sequencing products were separated by capillary electrophoresis in an automated sequencer (ABI 3130XL Genetic Analizer, Applied Biosystems) with a 36 cm length capillary and POP-7™ polymer, according to the manufacturer’s instructions. Data were analyzed with Sequencing Analysis Software version 5.3.1 (Applied Biosystems).

**Table 2:** Primer sequences and PCR amplification conditions for Sanger Sequencing (SS) validation

Gene	Chromosome Position	RefSeq	Coding DNA	Protein	PCR primers	Ta (°C)	Length amplicon (bp)
*CDKN2A*	chr9:21974792	NM_001195132	c.35delC	p.(Ser12TrpfsTer14)	F: ACTTCAGGGGTGCCACATTC	60	493
					R: GCGCTACCTGATTCCAATTC		
*TP53*	chr17:7579472	NM_000546.5	c.215C > G	p.Pro72Arg	F: TGAAGCTCCCAGAATGCCAG	60	136
					R: GCTGCCCTGGTAGGTTTTCT		
*TP53*	chr17:7577543	NM_000546.5	c.738G > A	p.Met246Ile	F: TGGCTCTGACTGTACCACCA	60	123
					R: CAAGTGGCTCCTGACCTGG		
*ERBB4*	chr2:212812278	NM_005235	c.298G > A	p.Glu100Ly	F: ACAGGCTACGTGTTAGTGGC	60	104
					R: GCCAAGGCATATCGATCCTCA		
*ERBB4*	chr2:212578373	NM_005235	c.884A > T	p.His295Leu	F: TGTTTTGAGCTTGTTTGCTGA	60	176
					R: GGGCAAATGTCAGTGCAAGG		
*ARID2*	chr12:46244997	NM_152641	c.3091C > T	p.Gln1031Ter	F: CGTCGTCCTCTACCCCTCAA	60	201
					R: CACCAGAGGCAGGCTGAC		
*KDR*	chr4:55972974	NM_002253	c.1416A > T	p.Gln472His	F: TACCATGGTAGGCTGCGTTG	60	191
					R: GGAAGTCCTCCACACTTCTCC		
*MET*	chr7:116340262	NM_001127500	c.1124A > G	p.Asn375Ser	F: ATTCTTTTCGGGGTGTTCGC	60	201
					R: TGGGGAACTGATGTGACTTACC		
*PIK3CA*	chr3:178927410	NM_006218	c.1173A > G	p.Ile391Met	F: AGGTGGAATGAATGGCTGAATTA	60	110
					R: ACCTCTTTAGCACCCTTTCGG		
*PPP6C*	chr9:127912080	NM_001123355	c.790C > T	p.Arg264Cys	F: GGTGACAGTATGGTCTGCTCC	60	148
					R: CGTTGTCGTTCTGGGAGGAA		
*BRAF*	chr7:140453136	NM_004333	c.1799 T > A	p.Val600Glu	F: GCTTGCTCTGATAGGAAAATGAGAT	60	175
					R: CATCCACAAAATGGATCCAGACAAC		
*BRAF*	chr7:140453136	NM_004333	c.1798_1799delGTinsAA	p.Val600Lys	F: GCTTGCTCTGATAGGAAAATGAGAT	60	175
					R: CATCCACAAAATGGATCCAGACAAC		
*BRAF*	chr7:140453135	NM_004333	c.1799_1800delTGinsAC	p.Val600Asp	F: GCTTGCTCTGATAGGAAAATGAGAT	60	175
					R: CATCCACAAAATGGATCCAGACAAC		
*BRAF*	chr7:140453145	NM_004333	c.1790 T > G	p.Leu597Arg	F: GCTTGCTCTGATAGGAAAATGAGAT	60	175
					R: CATCCACAAAATGGATCCAGACAAC		
*KIT*	chr4:55593464	NM_000222	c.1621A > C	p.Met541Leu	F: AGTGGCTGTGGTAGAGATCC	60	427
					R: CAAAAAGGTGACATGGAAAGC		
*NRAS*	chr1:115256529	NM_002524	c.182A > G	p.Gln61Arg	F: CACCCCCAGGATTCTTACAG	60	173
					R: TCCGCAAATGACTTGCTATT		
*NRAS*	chr1:115256530	NM_002524	c.181C > A	p.Gln61Lys	F: CACCCCCAGGATTCTTACAG	60	173
					R: TCCGCAAATGACTTGCTATT		
*PTEN*	chr10:89720709	NM_000314	c.860C > G	p.Ser287Ter	F: GCAACAGATAACTCAGATTGC	60	505
					R: TTCTTCATCAGCTGTACTCC		
*CDKN2A*	chr9:21971089	NM_001195132	c.256_268delGCCCGGGAGGGCT	p.Ala86fs	F: AGCTTCCTTTCCGTCATGC	60	0
					R: GGAAGCTCTCAGGGTACAAAT		

*Abbreviations*: *F* primer Forward; *R* primer reverse; *Ta* annealing temperature

### NGS concordance

The concordance of variant calls across the 3 different NGS approaches, was measured on with the Intra-class Correlation Coefficient (ICC) [32], using the IRR package within the R computational environment [33, 34]. The ICC analysis was calculated considering cut-off of 200 depth of coverage and VAF of 10.0%, and then repeated using only the VAF criterion.

## Results

The NGS analysis was performed using a specific multiple-gene panel constructed by the Italian Melanoma Intergroup, the IMI Somatic Panel, arranged in three primer pools, and designed using the Ion AmpliSeq Designer to explore the mutational status of selected regions (343 amplicons; amplicon range: 125–175 bp; coverage 100%) within the 25 genes reported as the most frequently mutated in melanomas by The Cancer Genome Atlas (TGCA) and successive NGS-based studies [5, 14].

### PGM™ ion torrent platform

**Fig. 1:**
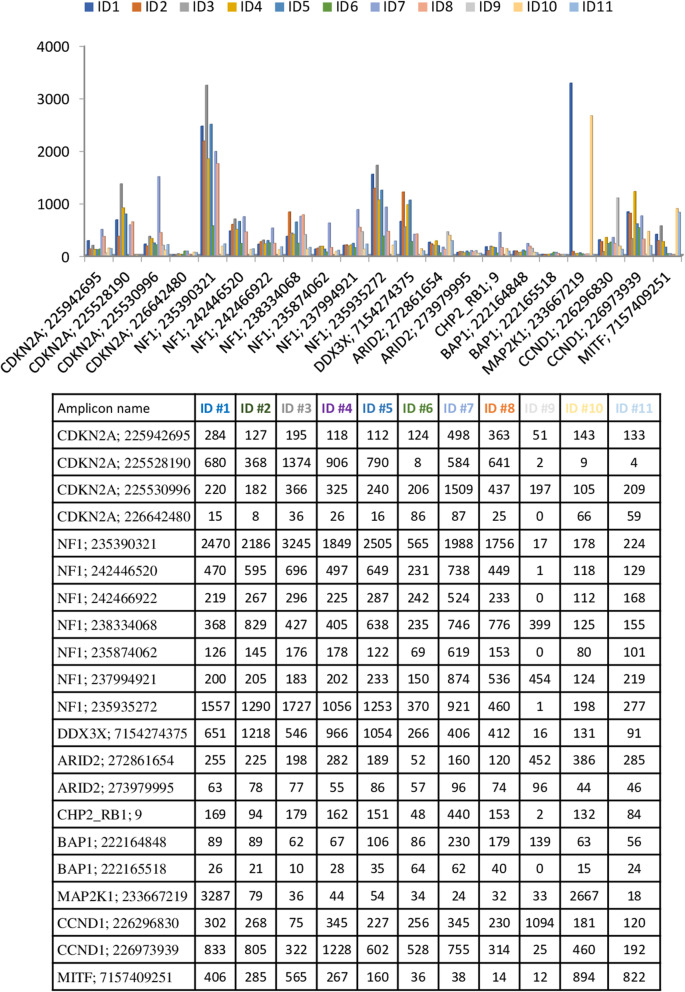
PGM™ platform low-covered regions. The figure shows for amplicons with a coverage lower than 200x in at least two tumor samples. The histograms report on the x axis the amplicons name not covered 200x in at least two sample for all case and in y axis the amplicon coverage

Eleven tumor samples were sequenced by IRCCS Ospedale Policlinico San Martino in Genoa on PGM™ Ion Torrent platform. The coverage and uniformity of each sample are reported in Additional File 1. The total number of reads was 12,475,778 (median average of 1,134,162 reads) with an average number of reads per amplicon and uniformity of 3023.7x and 87.6%, respectively. In these settings, more than 89.5% (ranging: 65.3–96.2%) of the targeted regions were covered at least 500x and 90.5% (ranging: 69.7–98.3%) of the targeted regions were covered 200x, and less than 4.0% (ranging: 1.5–26.5%) of targeted regions had coverage below 100x (Table 3a). Notably, the tumor sample with the highest number of amplicons not covered more than 200x was ID #9. More specifically, the sample ID #9 with a DIN 3.2 showed a 30.3% of amplicons <200x suggesting that low quality of gDNA could affect sequencing results. Low-covered regions (uncovered or with coverage <200x) in almost 2 tumor samples were constantly observed in 21/343 genes (≥18.2%; Fig. 1). In particular, 3 amplicons (AMPLP226642480, *CDKN2A*: chr9: 21974448–21,974,570; AMPLP273979995, *ARID2*: chr12: 46285681–46,285,772; AMPLP222165518, *BAP1*: chr3: 52443880–52,443,996) were never covered ≥200x.

**Table 3:** NGS data quality

		ID #1	ID #2	ID #3	ID #4	ID #5	ID #6	ID #7	ID #8	ID #9	ID #10	ID #11
**A**	N°Amplicons ≥500x	322	318	320	317	324	313	331	318	225	302	295
	% Amplicons ≥500x	93.9	92.7	93.3	92.4	94.5	91.3	96.5	92.7	65.6	88.0	86.0
	N°Amplicons ≥200x	337	334	332	336	334	331	338	335	240	327	328
	% Amplicons ≥200x	98.3	97.4	96.8	98.0	97.4	96.5	98.5	97.7	70.0	95.3	95.6
	N°Amplicons <100x	4	6	6	5	4	10	5	5	91	6	8
	% Amplicons <100X	1.2	1,7	1,7	1,5	1,2	2,9	1,5	1,5	26,5	1,7	2,3
	Average amplicon coverage	3,93	4002	3375	3,26	3522	3126	4083	3302	3714	2146	1737
	Uniformity (%)	90.1	89.8	88.2	91.15	91.44	82.3	92.5	90.7	65.1	91.1	91.8
**B**	N°Amplicons ≥500x	337	340	340	338	341	290	289	294	338	302	254
	% Amplicons ≥500x	98.3	99.1	99.1	98.5	99.4	84.5	84.3	85.7	98.5	88.0	74.1
	N°Amplicons ≥200x	342	342	342	342	343	330	332	338	339	331	324
	% Amplicons ≥200x	99.7	99.7	99.7	99.7	100.0	96.2	96.8	98.5	98.8	97.1	94.5
	N°Amplicons <100x	0	0	0	0	0	6	3	4	3	5	7
	% Amplicons <100x	0.0	0.0	0.0	0.0	0.0	1.7	0.9	1.2	0.9	1.5	2.0
	Average amplicon coverage	9,75	15,009	12,239	11,254	14,842	967	1137	1102	1647	1308	1083
	Uniformity (%)	94.7	92.7	93.9	96.2	93.9	95.6	96.0	97.1	92.0	95.7	93.8
**C**	N°Amplicons ≥500x	307	277	307	306	321	240	310	310	209	310	339
	% Amplicons ≥500x	89.5	80.8	89.5	89.2	93.6	70.0	90.4	90.4	60.9	90.4	98.8
	N°Amplicons ≥200x	336	326	336	335	337	300	336	334	286	334	340
	% Amplicons ≥200x	98.0	95.0	98.0	97.7	98.3	87.5	98.0	97.4	83.4	97.4	99.1
	N°Amplicons <100x	3	11	4	4	4	23	4	6	26	2	2
	% Amplicons <100x	0.9	3.2	1.2	1.2	1.2	6,7	1,2	1,7	7,6	0,6	0,6
	Average amplicon coverage	1,91	1267	1825	1617	2016	1531	2085	2292	1217	1387	3,73
	Uniformity (%)	93.9	93.3	93.0	94.2	95.6	81.2	92.7	91.3	80.5	94.2	96.2

The table shows for each NGS platforms ((**A**) PGM™ platform, (**B**) Proton™ platform, and (**C**) MiSeq™ Illumina platform) data quality for the eleven tumor samples in terms of uniformity (the percentage of bases in all target regions covered by at least 20% of the average base coverage depth reads), average amplicon coverage depth and number (%) of amplicons at different coverage

The VC plugin reported a total of 60 exonic genetic variants (51 Single Nucleotide Variants (SNVs), 2 Multi Nucleotide Variants (MNVs), and 7 frameshift deletions), irrespective of coverage and VAF (Additional File 2). Notably, all the *BRAF* mutations, previously detected by SS /Real Time PCR assay/Therascreen™ BRAF Pyro Kit, were confirmed in all tumor samples. In particular, eight tumor samples reported a *BRAF* mutation of which 5 was p.Val600Glu, 1 p.Leu597Arg, 1 p.Val600Lys, and 1 p.Val600Asp all sufficiently covered (>200x) with an VAF > 19.0% (Additional File 2). Among melanoma pharmacologically targetable genes, in addition to BRAF gene mutations, Ion Torrent called 2 *NRAS* mutations in different samples as follows: LRG_92/NM_002524.3: c.182A > G p.Gln61Arg and LRG_92/NM_002524.3: c.181C > A p.Gln61Lys with an VAF of 49.9% (6687x) and 64.6% (5642x), respectively. Notably, the two samples harboring *NRAS* mutation did not display mutations in *BRAF* gene supporting the idea that *BRAF* and *NRAS* mutations are commonly mutually exclusive.

### Proton™ ion torrent platform

**Fig. 2:**
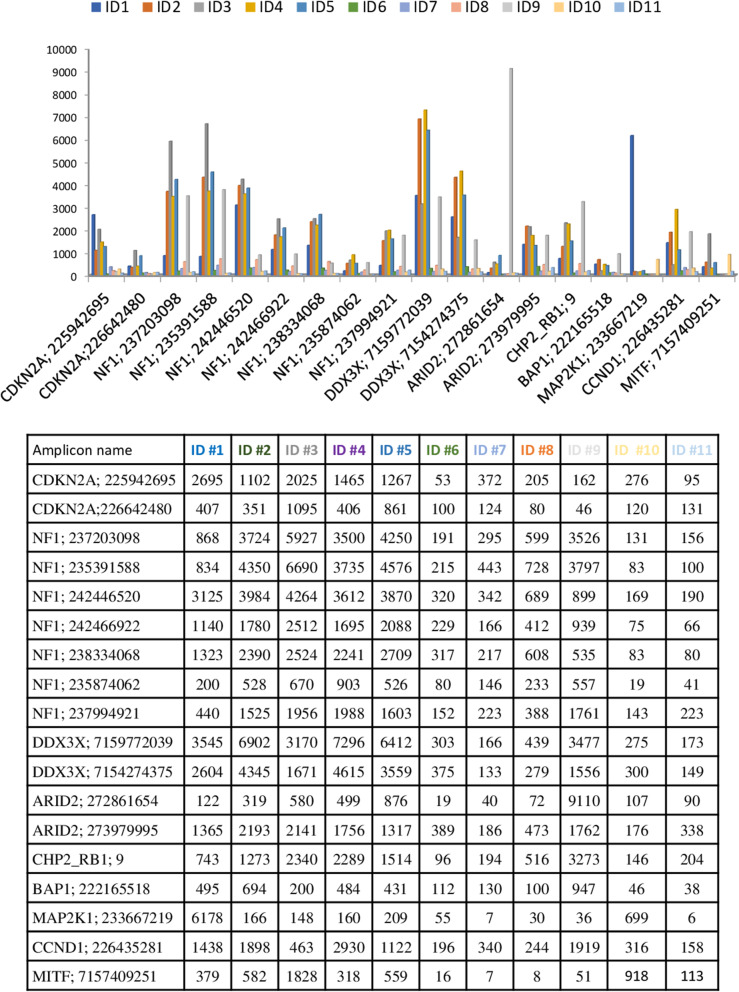
Proton™ platform low-covered regions. The figure shows amplicons with a coverage lower than 200x in at least two tumor samples. The histograms report on the x axis the amplicons name not covered 200x in at least two sample for all case and in y axis the amplicon coverage

The same eleven tumor samples were sequenced by the Unit of Cancer Genetics at the National Research Council (CNR) in Sassari on Proton™ Ion Torrent platform. The coverage and uniformity of each sample are reported in Additional File 1. The total number of reads was 25,637,162 (median average of 1,573,735 reads) with an average number of reads per amplicon and uniformity of 6748x and 94.7%, respectively. In these settings, more than 91.8% (ranging: 74.1–99.4%) of the targeted regions were covered at least 500x and 98.3% (ranging: 94.5–100.0%) of the targeted regions were covered 200x, and less than 0.74% (ranging: 0.0–2.0%) of targeted regions had coverage below 100x (Table 3b). The tumor sample with the highest number of amplicons not covered more than 200x was ID #11 with a 5.5% of amplicons <200x. However, the DIN of ID #11 sample was 6.6 which is a DNA good quality value. Low-covered regions (uncovered or with coverage <200x) in almost 2 tumor samples were constantly observed in 18/343 genes (≥5.2%; Fig. 2). Notably, 3 amplicons (AMPL-P233667219, MAP2K1: chr15:66735563–66,735,643; AMPL-P272861654, ARID2: chr12: chr12:46215132–46,215,226; AMPL-P226642480, CDKN2A: chr9:21994132–21,994,263; AMPL-P222165518) were not covered ≥200x in the half of samples. The NGS analysis reported a total of 78 exonic genetic variants (67 SNVs, 2 MNVs, and 9 Insertions/deletions (indels), irrespective of coverage and VAF (Additional File 3). All the 8 *BRAF* mutations disclosed by SS/Real Time PCR assay/Therascreen™ BRAF Pyro Kit were called in all tumor samples with a coverage >200x and an VAF > 18.8% (Additional File 3). In addition to *BRAF* gene mutations, Proton™ called 3 *NRAS* mutations in different samples: LRG_92/NM_002524.3: c.182A > G p.Gln61Arg, LRG_92/NM_002524.3: c.181C > A p.Gln61Lys, and LRG_92/NM_002524.3: c.35G > A p.Gly12Asp with an VAF of 47.8% (1987x), 64.6% (5642x), 5.3% (1958x), respectively. As above, the samples harboring *NRAS* mutations did not display mutations in *BRAF* gene.

### Illumina platform

**Fig. 3:**
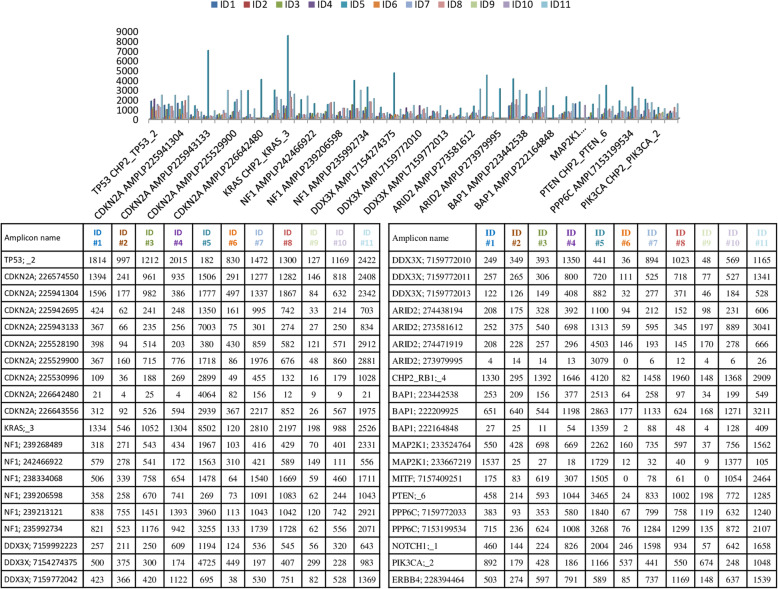
MiSeq™ Illumina platform low-covered regions. The figure shows amplicons with a coverage lower than 200x in at least two tumor samples. The histograms report on the x axis the amplicons name not covered 200x in at least two sample for all case and in y axis the amplicon coverage

The same series of tumor samples were sequenced by IRCCS Ospedale Policlinico San Martino in Genoa on Illumina MiSeq™ platform. The coverage and uniformity of each sample are reported in Additional File 1. The total number of reads was 7,562,830 (median average of 687,530 reads) with an average number of reads per amplicon and uniformity of 1897.6x and 89.3%, respectively. More than 85.8% (ranging: 60.9–98.8%) of the targeted regions were covered at least 500x and 95.4% (ranging: 83.4–99.1%) of the targeted regions were covered 200x, and less than 8.1% (ranging: 2–26%) of targeted regions had coverage below 100x (Table 3c). The ID #9 was the sample with the highest number of amplicons not covered more than 200x (16.6% of amplicons with coverage <200x). A total of 40 amplicon regions (11.6%; Fig. 3) in almost 2 tumor samples were present with a coverage <200x. Seven amplicons (AMPL-P225530996, CDKN2A: chr9: 21974673–21,974,792; AMPL-P226642480, *CDKN2A*: chr9: 21974448–21,974,570; AMPL-7159772013, *DDX3X:* chrX:41206085–41,206,199; AMPL-P273705807, *ARID2*: chr12:46285693–46,285,805; AMPL-P222164848*, BAP1*: chr3:52443752–52,443,884; AMPL-P233667219, *MAP2K1:* chr15:66735563–66,735,643; AMPL-7157409251, *MITF:* chr3:70013925–70,014,246) were observed not covered ≥200x in the half of samples. The DNA Amplicon App on BaseSpace displayed a total of 83 exonic genetic variants (64 SNVs, 2 MNVs, and 17 indels) (Additional File 4).

The exon 15 of *BRAF* gene was sufficiently covered (>200x) reporting the 8 *BRAF* mutations previously disclosed by SS/Real Time PCR assay/Therascreen™ BRAF Pyro Kit and 2 *NRAS* mutations in 2 different samples confirmed by SS (LRG_92/NM_002524.3: c.182A > G p.Gln61Arg with an VAF of 51.7% and coverage of 1657x; LRG_92/NM_002524.3: c.181C > A p.Gln61Lys with an VAF 62.2% and coverage of 623x) (Additional File 4).

### Analytical performance

We evaluated the performance of somatic variants detection by three NGS platform using the 11 tumor samples that had been blindly sequenced in the two centers.

**Fig. 4:**
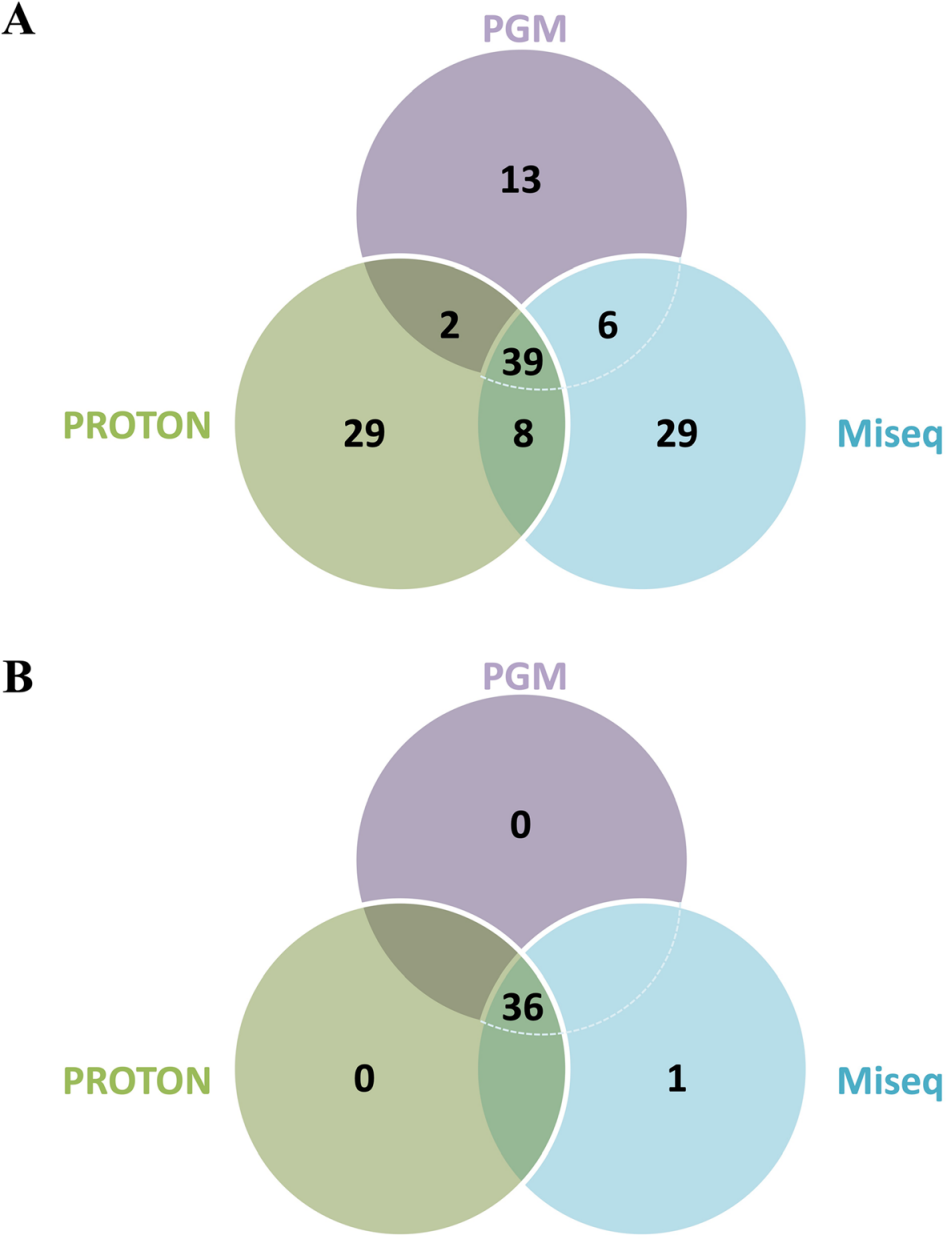
Venn Diagram of 126 exonic genetic variants called using the three different NGS platforms regardless of coverage and allele frequency (**a**) and of 37 exonic genetic variants called using the three different NGS platforms with an VAF > 10% (**b**)

The combination of variant calls between the three platforms identified a total of 126 exonic genetic variants among the different systems irrespective of coverage and VAF (Additional File 5**;** Fig. 4a). By setting a coverage ≥200x and VAF ≥10%, a total of 36 variants were called by the three systems (PGM™, Proton™, and Miseq™) (Table 4). Therefore, concordance was calculated based on our assay detection limit (coverage ≥200x and VAF ≥10%) on these 36 variants. Despite different coverage depending on the platform used and pipeline of analysis, considering a minimum coverage of 200x and a VAF greater than 10%, the concordance on the absolute number of exonic variants found by each of the three NGS assays was 100%. Moreover, all variants with frequency higher than 10% were confirmed and validated by SS. In general, similar VAF were reported across the three platform for the 36 genetic variants, with an ICC of 0.901 (95%CI: 0.837–0.945, *p* < 0.01). The allele frequencies between the two Ion Torrent platforms displayed an ICC of 0.868 whereas ICC between PGM versus Illumina was 0.979 and ICC between Proton versus Illumina was 0.842. Only for three variants Proton called very dissimilar allele frequency compared to the other two NGS systems (±25.5). Noteworthy, Illumina called two additional unique CDKN2A variants (NM_001195132: c.35C > T (p.Ser12Leu) and c.35delC (p.Ser12TrpfsTer14)) in one tumor sample (ID #10), but both variants had a coverage of 108x and were thus excluded by our detection limit (Additional File 4).

**Table 4:** Variants called by the three NGS systems with a coverage of at least 200x and a VAF ≥10%

Gene	RefSeq	Protein	DNA change	N°
*ARID2*	NM_152641	p.Gln1031Ter	c.3091C > T	1
*BRAF*	NM_004333	p.Leu597Arg	c.1790 T > G	1
*BRAF*	NM_004333	p.Val600Glu	c.1799 T > A	5
*BRAF*	NM_004333	p.Val600Lys	c.1798_1799delGTinsAA	1
*BRAF*	NM_004333	p.Val600Asp	c.1799_1800delTGinsAC	1
*CDKN2A*	NM_001195132	p.Ala86fs	c.256_268delGCCCGGGAGGGCT	1
*ERBB4*	NM_005235	p.Glu100Lys	c.298G > A	1
*ERBB4*	NM_005235	p.His295Leu	c.884A > T	1
*KDR*	NM_002253	p.Gln472His	c.1416A > T	4
*KIT*	NM_000222	p.Met541Leu	c.1621A > C	2
*MET*	NM_001127500	p.Asn375Ser	c.1124A > G	1
*NRAS*	NM_002524	p.Gln61Arg	c.182A > G	1
*NRAS*	NM_002524	p.Gln61Lys	c.181C > A	1
*PIK3CA*	NM_006218	p.Ile391Met	c.1173A > G	2
*PPP6C*	NM_001123355	p.Arg264Cys	c.790C > T	1
*PTEN*	NM_000314	p.Ser287Ter	c.860C > G	1
*TP53*	NM_000546	p.Pro72Arg	c.215C > G	10
*TP53*	NM_000546	p.Met246Ile	c.738G > A	1

Interestingly, the two *CDKN2A* genetic variants started in the same chromosome position with a considerable different VAF. Since one of the two had been called by Illumina with a VAF of 48.1%, we decided to validate it by SS. The SS confirmed the presence in this chromosome position (NM_001195132: chr9:21974792) of p.(Ser12TrpfsTer14) with a VAF ~ 50% instead of p.Ser12Leu. A possible explanation of the incorrect call could be the position of the variant (GRCh37.p13; chr9:21974792) located in the last base of the designed amplicon. The region in which Illumina called the *CDKN2A* variant was covered at a similar (105X) and higher (250X) depth by PGM™ and Proton™, and therefore we considered this variant as called at a frequency of 0% by these two platforms. In light of this findings, we re-assessed the concordance between the three platforms dropping the coverage cut-off and including all the 37 variants with VAF higher than 10% (Fig. 4b), and obtained an ICC of 0.863 between the three platforms (95%CI = 0.779–0.922, *p* < 0.01).

## Discussion

As the number of actionable genes in melanoma tumors is steadily growing there is an increasing need to perform multi-gene mutation testing in molecular diagnostics. Several NGS panels are commercially available, but these panels often contain genes or hotspots that are not of particular interest for molecular diagnostics due to their uncertain clinical significance, or to the lack of genes or hotspots specific for tumor types studied. Today, only two commercial NGS panels are specifically designed to test somatic melanoma. However, these panels, namely Sentosa® SQ Melanoma Panel (Vela Diagnostics) and MELP Panel (MAYO Clinic Laboratories), contain only 10 (16 exons) and 5 (17 exons) genes, respectively, thus leaving out several genes of interest in the cutaneous melanoma research area. To overcome this issue, we have developed a custom panel to screen hotspots in 25 genes for clinically relevant mutations in melanoma based on the available literature at the time of panel design, including information retrieved from TGCA and available literature data on melanoma. The relevant factors taken into consideration when selecting the regions of interest to be included in the panel were the presence of variants with clinical significance in terms of prognostic, therapeutic and diagnostic value and the estimated cost per sample with an optimal depth of coverage. In particular, our custom panel covers all regions of MELP Panel (MAYO Clinic Laboratories), while it does not include *AKT3* (exon 5 and 6) and *FGFR3* (exon 7, 9, and 14) genes included in the Sentosa® SQ Melanoma Panel (Vela Diagnostics). However, *FGFR3* activating mutations play a key role in the pathogenesis of bladder cancer and have been found in benign conditions such as seborrheic keratosis and epidermal nevi. Moreover, TCGA cutaneous *melanoma* project has revealed low-frequency pathogenetic mutations in *AKT3 (0.3%)* and *FGF3 (2.5%)* (Cancer Genome Atlas Network, 2015). However, it should be observed that currently only *BRAF* exon 15 testing, and partially, *NRAS* exons 2 and 3, and *KIT* exons 11 and 13, in *BRAF* negative cases is recommended in clinical routine for the selection of target therapy and/or inclusion in clinical trials, and all these exons are included in the three panels here discussed. The application of the panel here described is for research purposes. The panel has already been used in research studies performed within the Italian Melanoma Intergroup with the analyses performed in a single center [30].

We therefore obtained a panel with a total size of 35.13 kb, made up of three primers pools and with limited amount of DNA required (30 ng), offering sufficiently extensive and clinically relevant mutational profiling in a cost-efficient way. We then evaluated the concordance of this custom NGS panel in the identification of somatic genetic variants clinically relevant in melanoma patients using three different benchtop sequencers by a bicentric-study. To do this, we tested the panel using the most used NGS platform available in the laboratories: Ion Torrent PGM™ and Ion Proton™ for the ThermoFisher and MiSeq™ benchtop sequencers for the Illumina. Notably, at the time of the “IMI somatic panel” design the Ion Torrent S5 XL sequencer (ThermoFisher Scientific) was not present in the two centers, for the evaluation on this additional NGS platform, so due to the limited availability of the DNA of the eleven samples of the study, another patients setting was subsequently tested on S5 XL. In any case, the S5 XL sequencer employs the same chemistry as the Ion Torrent PGM™ and the Ion Torrent Proton™, so would not be relevant to our analysis. In fact, although several platforms available for routine diagnostic applications can perform high-throughput analysis within few days, with considerably reduced costs compared to SS [35], two of these are mainly used in clinical laboratories: Ion Torrent and Illumina systems.

We also estimated the total cost for the analysis of a single patient with the “IMI somatic panel” using the three different sequencing platforms. The cost for testing 25 genes using the “IMI somatic panel” was €270 (loading 3 samples on chip 316v2), €337 (loading all samples on Miseq Reagent Nano kit v2), and €398 (loading all samples on Ion PI Chip Kit V2) per sample for PGM™, Illumina, and Proton™, respectively, not taking into account panel primers, DNA extraction and quantity/quality control, labor time and bioinformatics analysis costs.

All platforms used in this study demonstrated comparable performance in the detection of somatic variants from the DNA samples tested, reaching an amplicon mean coverage higher than 1897x and an uniformity average greater than 87.6%. The Proton™ platform has revealed to have higher NGS quality metrics compared to the other 2 platforms. This data could be due to a load of fewer samples, which allowed to obtain a superior coverage than that of the other platforms.

Our analysis revealed that some amplicons are consistently not covered >200x across all samples and NGS platforms. Of note, two amplicons (CDKN2A-226,642,480 and MAP2K1–233,667,219) have been constantly covered less than 200x in half of the samples analyzed, proving that some amplicons in the “IMI somatic panel” design have an intrinsic impairment in their coverage ability. Published scientific data have shown how uneven coverage of amplicons is associated with GC bias introduced during PCR amplification of library, cluster amplification, or sequencing. In fact, the GC content of the amplified region is also critical for NGS sequencing performance on both Illumina and Ion Torrent platforms [36–40]. However, only the CDKN2A-226,642,480 amplicon displayed % GC content higher than 90, explaining a lower coverage, while the MAP2K1–233,667,219 amplicon showed a % GC of 33 [39]. Moreover, not even the amplicons length can explain this lack of coverage, since the “IMI somatic panel” designed has an amplicon range of 125-175 bp.

Finally, gDNA degradation status also did not influence the NGS quality data since the three different NGS platforms showed a different coverage for the same sample analyzed (Additional File 1), irrespective of DIN, although unsurprisingly, the DIN values were lower in FFPE compared to fresh frozen samples. On the contrary, some amplicons show consistently a coverage <200x across all samples and NGS platforms, regardless of sample DIN.

Regardless the NGS quality metrics, the three NGS platforms achieved a very good concordance (ICC of 0.901; 95%CI: 0.837–0.945, *p* < 0.01) considering a 200 depth of *coverage* and a *VAF* of 10.0%. It is known that Ion torrent NGS platforms present a higher per base error rate and a quality of base calling accuracy lower than that of Illumina sequencing platforms. Moreover, the Ion torrent platforms have a tendency of misreading the length of homopolymers compared to other platforms (e.g. Illumina) [36, 37, 41]. Unlike the two Ion Torrent platforms, in one tumor sample the Illumina platform called two different genetic variants [NM_001195132: c.35C > T (p.Ser12Leu) and c.35delC (p.Ser12TrpfsTer14)] in the same position of a *CDKN2A* amplicon (AMPL-225530996). Although the coverage of the aforementioned *CDKN2A* amplicon was similar across the three different platforms (105x, 230x and 108x for the PGM™, Proton™ and Illumina sequencer, respectively), the two variants were only called by the Illumina platform. Interestingly, the p.(Ser12TrpfsTer14) *CDKN2A* variant was confirmed by SS at a VAF of around 50.0%.

Considering this *CDKN2A* additional variant called by Illumina, the ICC between the three platforms remains good (ICC of 0.863; 95%CI = 0.779–0.922, *p* < 0.01).

A possible explanation of this phenomenon could be due to the well documented characteristic of the Ion Torrent’s current semiconductor sequencing platforms to call a higher number of indel error rate, particularly after long homopolomeric stretches, compared to Illumina platforms [41, 42]. In fact, Illumina’s overall indel error rate is the lowest of all NGS technologies. Moreover, paired-end reads sequencing is more sensitive and accurate than single-end reads sequencing, because it greatly facilitates alignment operations, allowing among other things, to detect any deletion, duplication or insertion in the patient’s DNA. The reason why Illumina miscalled the variant and identified it as SNV at a frequency of around 50% could be clarified by the fact that the genetic variants were both located at the end of the amplicon AMPL-225530996. The risk of false negative variants, as well as the allele drop-out phenomenon could be reduced by a tiling primer design that results in multiple overlapping amplicons for each target, to ensure the correct identification of all variants present in the target regions of the panel design. Moreover, this bias could be solved decreasing the number of samples sequenced in the same NGS run, which will increase coverage per sample while deliver a raised cost per sample for sequencing. Specific regions refractory to NGS, such as AMPL-225530996, need to be sequenced by SS and/or validated by alternative assays, in order to cover the gap and to validate the NGS data [43].

All these observations justify the need to improve analytical solutions to detect somatic mutations with high confidence, to avoid false positives or inaccurate call measurements. Nevertheless, both the detection of some variants located at the end of the amplicons mistakenly called and the insufficiently coverage highlighted the importance of validating variants by an independent test before clinical application. Moreover, NGS results should not be transferred to clinical reports and practice without acceptable validation. It is fundamental to confirm the genetic variation on a newly extracted DNA from the same sample using another NGS platform, SS, or another proper technique, in order to exclude false positive results. Indeed, in our study, all variants called at VAF higher than 10% were further confirmed by SS (Table 2). Moreover, all samples were previously screened for the presence of mutations in *BRAF* codon 15 by Real Time PCR assay (PNAClamp™ BRAF Mutation Detection Kit; Panagene, Daejeon, Korea) and Therascreen™ BRAF Pyro assay (Qiagen, Valencia, CA) (Table 1). In fact, PNAClamp™ and Therascreen™ tests were performed as part of the routine diagnostic approach and the outcome of these tests was documented in the patient report file and communicated with the medical oncologists. The technique used to validate the results should be included in the NGS report. Finally, all variants should be annotated and reported according to the HGVS [44] and, for diagnostic purposes, only those genes with an established (i.e. published and confirmed) relationship between the aberrant genotype and melanoma should be included in the analysis. The information provided in the NGS report should be limited to the disease status, its targets, the names of the genes tested, their reportable ranges, as well as the analytical sensitivity and specificity of the technique [45, 46]. On the contrary, variants not linked with melanoma or gene variants not requested by medical oncologist should be not reported. It should also be emphasized that the interpretation of pathogenicity of a variant must be circumscribed to the evidence of its role in melanoma tumorigenesis at the time of the report, and that it could change over time as new information becomes available.

Massive efforts should be made to unify the interpretation and reporting of NGS molecular results among laboratories. In this context, a joint consensus recommendation for the interpretation and reporting of sequence variants in cancer was published [44].

The IMI somatic panel represent a relevant, highly scalable, and robust tool that is easy to implement and that can be fully adapted to daily clinical practice in determining melanoma actionable gene mutations, with a very good concordance - to detect somatic variants with frequencies higher than 10% with a coverage of 200x among the three NGS platforms. However, further validation studies on a greater number of samples from metastatic melanoma patients are required. Currently, the screening of clinically-actionable mutations is performed on FFPE tumor biopsies, but the amount of tumor tissue is often limited, and DNA quality may not be always optimal. We showed that this panel can be applied in the analysis of tumor FFPE tissue with varying status of DNA degradation. In fact, for all the samples, gDNA obtained from routine molecular testing of *BRAF* in metastatic melanoma and extracted with different methods in the two laboratories proved to be good reference material for the evaluation of this panel.

## Conclusions

Since the advent of targeted therapy, treatment decisions are increasingly based on the molecular features of the tumor. Hence, laboratories need comprehensive molecular testing covering all actionable melanoma mutations using only limited amount of tumor tissue, mostly FFPE tissues, in a time-and cost-effective manner and with good performance. We show that the IMI panel, which include all established and several candidate melanoma driver genes, has optimal concordance- in the detection of actionable melanoma mutations using the main three NGS platforms available in research and clinical centers. We also achieve a good sequencing performance based upon amplicon and hotspot variants within the 25 genes of our designed NGS custom panel, obtaining an average amplicon coverage above 1800x with all three platforms.

Although our study is limited by the small number of samples analyzed, our study showed a high level of concordance in mutational patterns of the panel between two centers, using different extraction methods and NGS platforms to identify challenges and opportunities of center-specific platforms/protocols to analyze the same samples with the same panel. To the best of our knowledge, this is the first study in which concordance obtained using an NGS melanoma custom panel was evaluated by a bi-centric study with three different NGS platforms. This study may lay the ground for developing collaborations and share positive controls here analyzed to other centers working together within the Italian Melanoma Intergroup.

## Supplementary Information

**Additional file 1.** NGS metrics. The table shows the sequencing metrics detected from the three NGS platform for each sample. Each column reported the number of read per amplicon of each sample. Coverage lower than 200x is indicated in bold.

**Additional file 2. **List of exonic genetic variants called by PGM™ VC for the eleven tumor samples. All variants are annotated with the gene ID and locus RefSeq, and the mutation nomenclature is based on the convention recommended by the Human Genome Variation Society (http://www.hgvs.org/mutnomen/) other than the variant allele and the nature of the allele call (heterozygous or homozygous). Frequency data indicate the percentage of the variant allele detected by PGM VC. Moreover, they are annotated for dbSNP (rs number) or COSMIC v86 database, together with FATHMM score. The FATHMM is a functional score for individual mutations from FATHMM-MKL are in the form of a single *p*-value, ranging from 0 to 1. Scores above 0.5 are deleterious, but in order to highlight the most significant data in COSMIC, only scores ≥0.7 are classified as ‘Pathogenic’ whereas mutations are classed as ‘Neutral’ if the score is ≤0.5 [47]. The “Effect” column reports the effect of nucleotide change on the protein. The last three columns of the table report the GnomAD Frequency, the predictive effect on the protein based on SIFT, and the conservation score, namely GERP. Converted rankscore is reported for SIFT. To obtain the rankscore, Sorting Intolerant from Tolerant (SIFT) scores were first converted to SIFTnew = (1-SIFTori), then ranked among all SIFTnew scores in dbNSFP. The rankscore is the ratio of the rank the SIFTnew score over the total number of SIFTnew scores in dbNSFP. If there are multiple scores, only the largest (most damaging) rankscore is presented. Rankscores range from 0.02654 to 0.87932. Genomic Evolutionary Rate Profiling (GERP) is a conservation score calculated by quantifying substitution deficits across multiple alignments of orthologues using the genomes of 35 mammals. It ranges from − 12.3 to 6.17, with 6.17 being the most conserved [48]. *Abbreviations:* VC: Variant Caller; −: no available data; GERP: genomic evolutionary rate profiling. SIFT: Sorts Intolerant From Tolerant. GnomAD: Genome Aggregation Database

**Additional file 3. **List of exonic genetic variants called by Proton™ VC for the eleven tumor samples. All variants are annotated with the gene ID and locus RefSeq, and the mutation nomenclature is based on the convention recommended by the Human Genome Variation Society (http://www.hgvs.org/mutnomen/) other than the variant allele and the nature of the allele call (heterozygous or homozygous). Frequency data indicate the percentage of the variant allele detected by Proton VC. Moreover, they are annotated for dbSNP (rs number) or COSMIC v86 database, together with FATHMM score. The FATHMM is a functional score for individual mutations from FATHMM-MKL are in the form of a single *p*-value, ranging from 0 to 1. Scores above 0.5 are deleterious, but in order to highlight the most significant data in COSMIC, only scores ≥0.7 are classified as ‘Pathogenic’ whereas mutations are classed as ‘Neutral’ if the score is ≤0.5 [47]. The “Effect” column reports the effect of nucleotide change on the protein. The last three columns of the table report the GnomAD Frequency, the predictive effect on the protein based on SIFT, and the conservation score, namely GERP. Converted rankscore is reported for SIFT. To obtain the rankscore, Sorting Intolerant from Tolerant (SIFT) scores were first converted to SIFTnew = (1-SIFTori), then ranked among all SIFTnew scores in dbNSFP. The rankscore is the ratio of the rank the SIFT new score over the total number of SIFTnew scores in dbNSFP. If there are multiple scores, only the largest (most damaging) rankscore is presented. Rank scores range from 0.02654 to 0.87932. Genomic Evolutionary Rate Profiling (GERP) is a conservation score calculated by quantifying substitution deficits across multiple alignments of orthologues using the genomes of 35 mammals. It ranges from − 12.3 to 6.17, with 6.17 being the most conserved [48]. *Abbreviations:* VC: Variant Caller; −: no available data; GERP: Genomic Evolutionary Rate Profiling. SIFT: Sorts Intolerant From Tolerant. GnomAD: Genome Aggregation Database.

**Additional file 4. **List of exonic genetic variants called by MiSeq™ Illumina Variant interpreter for the eleven tumor samples. All variants are annotated with the gene ID and locus RefSeq, and the mutation nomenclature is based on the convention recommended by the Human Genome Variation Society (http://www.hgvs.org/mutnomen/) other than the variant allele and the nature of the allele call (heterozygous or homozygous). Frequency data indicate the percentage of the variant allele detected by Illumina. Moreover, they are annotated for dbSNP (rs number) or COSMIC v86 database, together with FATHMM score. The FATHMM is a functional score for individual mutations from FATHMM-MKL are in the form of a single p-value, ranging from 0 to 1. Scores above 0.5 are deleterious, but in order to highlight the most significant data in COSMIC, only scores ≥0.7 are classified as ‘Pathogenic’ whereas mutations are classed as ‘Neutral’ if the score is ≤0.5 [47]. The “Effect” column reports the effect of nucleotide change on the protein. The last three columns of the table report the GnomAD Frequency, the predictive effect on the protein based on SIFT, and the conservation score, namely GERP. Converted rankscore is reported for SIFT. To obtain the rankscore, Sorting Intolerant from Tolerant (SIFT) scores were first converted to SIFTnew = (1-SIFTori), then ranked among all SIFTnew scores in dbNSFP. The rankscore is the ratio of the rank the SIFTnew score over the total number of SIFTnew scores in dbNSFP. If there are multiple scores, only the largest (most damaging) rankscore is presented. Rankscores range from 0.02654 to 0.87932. Genomic Evolutionary Rate Profiling (GERP) is a conservation score calculated by quantifying substitution deficits across multiple alignments of orthologues using the genomes of 35 mammals. It ranges from − 12.3 to 6.17, with 6.17 being the most conserved [48]. *Abbreviations:* VC: Variant Caller; −: no available data; GERP: Genomic Evolutionary Rate Profiling. SIFT: Sorts Intolerant From Tolerant. GnomAD: Genome Aggregation Database.

**Additional file 5. **Coding genetic variants called by all platforms for the eleven tumor samples. *Abbreviations:* VC: Variant Caller; −: no available data; GERP: Genomic Evolutionary Rate Profiling. SIFT: Sorts Intolerant From Tolerant. GnomAD: Genome Aggregation Database.

## Data Availability

The dataset used and/or analysed in the current study are available from the corresponding author on reasonable request.

## Abbreviations

AIOM: Italian Association of Medical Oncology
CI: Confidence interval
CNR: National research council
DIN: DNA integrity number
EMQN: European Molecular Genetics Quality Network
EQA: External quality assessment
FFPE: Formalin-fixed paraffin-embedded
gDNA: Genomic DNA
ICC: Intraclass correlation coefficient
indels: Insertions/deletions
MNVs: Multi nucleotide variants
NGS: Next-generation sequencing
PNA: Peptide nucleic acid
SNVs: Single nucleotide variants
SS: Sanger sequencing
TGCA: The cancer genome atlas
VAF: Variant allele frequency
VC: Variant caller
WES: Whole exome sequencing
WGS: Whole genome sequencing
